# A Case of Triplets—Saccharated Calomel

**Published:** 1873

**Authors:** A. S. Whitaker

**Affiliations:** Palmetto, Ga.


*Messrs. Editors*,—As possibly a matter of some interest, I will state that I was called to see Mrs.——, aged thirty-six years, in her sixth pregnancy. I found her with the usual symptoms of natural labor, which lasted about six hours. She was delivered of three children. There was an interval of half an hour between the birth of each. All presented with breech, and each enveloped in a separate sack. The two first were girls, and the third a boy. They lived about four hours, and died, about half an hour apart, as they were born. Seven months children. The mother recovered without any unusual symptoms.

A. S. Whitaker, M. D.

*Palmetto, Ga., May*, 1873.

---

Saccharated Calomel.—In our August number, we published a paper upon saccharated calomel, in which the writer was unable to state positively to whom was due the honor of having introduced to the profession this valuable combination.

We are happy to be able now to state, upon the authority of the originator himself, that this honor is justly due to Dr. John M. Johnson, of Atlanta, Ga.

We are likewise informed that the Doctor has yet other novelties of value in reserve, which he has promised to give to the public through our columns, and which we hope soon to present.

